# The Effects of Trimetazidine on QT-interval Prolongation and Cardiac Hypertrophy in Diabetic Rats

**DOI:** 10.5935/abc.20180248

**Published:** 2019-02

**Authors:** Fatemeh Ramezani-Aliakbari, Mohammad Badavi, Mahin Dianat, Seyed Ali Mard, Akram Ahangarpour

**Affiliations:** Physiology Research Center and Department of Physiology, Faculty of Medicine, Ahvaz Jundishapur University of Medical Sciences, Ahvaz - Iran

**Keywords:** Diabetes Mellitus, Trimetadizine, Cardiomegaly, Electrocardiology, Oxidative Stress, Rats

## Abstract

**Background:**

Trimetazidine (TMZ) is an anti-ischemic drug. In spite of its protective effects on cardiovascular system, there is no scientific study on the usefulness of TMZ treatment for prolonged QT interval and cardiac hypertrophy induced by diabetes.

**Objectives:**

To evaluate the effects of TMZ on QT interval prolongation and cardiac hypertrophy in the diabetic rats.

**Methods:**

Twenty-four male Sprague-Dawley rats (200-250 g) were randomly assigned into three groups (n = 8) by simple random sampling method. Control (C), diabetic (D), and diabetic administrated with TMZ at 10 mg/kg (T10). TMZ was administrated for 8 weeks. The echocardiogram was recorded before isolating the hearts and transfer to a Langendorff apparatus. Hemodynamic parameters, QT and corrected QT interval (QTc) intervals, heart rate and antioxidant enzymes were measured. The hypertrophy index was calculated. The results were evaluated by one-way ANOVA and paired t-test using SPSS (version 16) and p < 0.05 was regarded as significant.

**Results:**

The diabetic rats significantly indicated increased hypertrophy, QT and QTc intervals and decreased Left ventricular systolic pressure (LVSP), Left ventricular developed pressure (LVDP), rate pressure product (RPP), Max dp/dt, and min dp/dt (±dp/dt max), heart rate, superoxide dismutase (SOD), glutathione peroxidase (GPx) and catalase in the heart. Treatment with TMZ in the diabetic animals was significantly improved these parameters in comparison to the untreated diabetic group.

**Conclusions:**

TMZ improves QTc interval prolongation and cardiac hypertrophy in diabetes.

## Introduction

Diabetes is associated with cardiovascular disorders and increased mortality rate in diabetic patients.^1^ The statistic reveals that 30 million people were suffered from diabetes worldwide in 1985 and recently, it is predicted by WHO, there will be 300 million by the year 2025.^2^

Diabetic cardiomyopathy is known as the structural and functional alterations in the heart induced by diabetes that are associated with cardiac hypertrophy, diastolic and/or systolic dysfunction in the absence of hypertension, valvular and ischemic heart diseases and other cardiac disorders.^3,4^

QT and QTc intervals are electrocardiographic parameters that regarded as critical predictors of mortality and stroke in diabetic patients.^5,6^ The pathological QT prolongation is known as a risk factor that increases ventricular arrhythmias and other heart diseases. Moreover, ventricular hypertrophy plays an important role in developing prolonged QT interval-related diabetes.^7^ A previous study has confirmed the negative effects of hypertrophy and QT interval prolongation on the function of heart in diabetes.^8^ The homeostasis of energy is effective in decreasing the hypertrophy in the heart.^9^

Trimetazidine (TMZ) is an anti-angina agent that is known to improve metabolism of energy in the heart subjected to ischemia.^10,11^ Previous studies have indicated reduced fatty acid oxidation via reducing mitochondrial 3-ketoacyl CoA thiolase (3-KAT) activity in beta-oxidation by TMZ treatment.^12^ Others also indicated that TMZ has protective effects on cardiac fibrosis resulted from pressure overload.^13^ In addition, there are some other investigations showing that the treatment with TMZ has positive effects on cardiac function in diabetic individuals with cardiovascular disorders.^14^ Taken together, these results from related studies make evidence that TMZ has beneficial effects on cardiovascular system. However, the role of TMZ in QT interval prolongation and cardiac hypertrophy improvement in diabetes was still unknown. Therefore, the present study was undertaken to evaluate the effects of TMZ on QT interval prolongation and cardiac hypertrophy in the diabetic animals.

## Methods

### Chemical

Trimetazidine (TMZ), heparin and alloxan were obtained from Sigma Chemical Co. (St. Louis, MO, U.S.A.) and Ketamine and xylazine purchased from Alfasan Co (Woderen- Holland).

### Animal

Twenty-four adult male Sprague-Dawley rats (250 ± 20 g) were housed under standard conditions (20 ± 5°C, 12-hour light/dark cycle, and free available to water and food) during the study period. All the experimental protocols followed the Consensus Author Guidelines on Animal Ethics and Welfare and the national guidelines for conducting animal studies (Ethics Committee permission No. APRC-94-25 Ahvaz Jundishapur University of Medical Sciences, Ahvaz, Iran).^15^

The sample size of each group was computed to be eight by the formula:^16^

$$\begin{array}{c} n=\frac{{\left(Z_{1-\alpha /2}+Z_{1-\beta }\right)}^{2}\times \left(S_{1}^{2}+S_{2}^{2}\right)}{d^{2}}=\\ \frac{{\left(1.96\times 1.29\right)}^{2}\times \left(13.52^{2}+9.07^{2}\right)}{{\left(89-70\right)}^{2}}=7.75∼8 \end{array}$$

where S_1_^2^ and S_2_^2^ are means.

The animals were randomly divided into three groups (n = 8) by simple random sampling method. Control (C), diabetic (D) and diabetic administrated with TMZ at 10 mg/kg (T10).^17^ TMZ was treated orally by gavage once daily for 8 weeks.

### Diabetic model

Diabetes was induced by intraperitoneal administration of alloxan at 120 mg/kg. After 6 h, the animals were orally treated with 10% glucose solution (10 mL). They were further kept for 24 h on 5% glucose solution to reduce fatal hypoglycemic resulted from alloxan. The rats, indicating fasting blood glucose ≥ 250 mg/dL, reduced body weight, dyslipidemia, increased hepatic enzymes and clear signs of polyuria, polyphagia and polydipsia after 4 days were regarded as diabetic animals and used for the experiment.^18^

### Electrocardiography

The animals were anesthetized by heparin, ketamine and xylazine (1000 U/kg, 50, and 5 mg/kg, respectively), lead II was recorded by Bio Amp and controlled using a Power Lab system (AD Instruments, Australia). QT interval and heart rate were measured. Corrected QT interval (QTc) was calculated by Bazett formula normalized as QTc = QT/(RR/*f*)^1/2^, where RR is R-R interval and *f* = 150 ms.^19,20^

### Isolation of hearts

After echocardiogram (ECG) recording, the cannulation and ventilation of trachea were performed using an animal ventilator (UGO BASILE, model: 7025). The cannulation of aorta was carried out by a central incision in the aorta. The hearts were conveyed to the Langendorff system. The perfusion of heart was carried out by Krebs-Henseleit solution (5% carbon dioxide and 95% oxygen, 37°C, pH = 7.4, 8 ml/min). A latex balloon was inserted in the left ventricle for the measurement of left ventricular pressure (LVP) by Power Lab system (AD Instruments, Australia). Left ventricular end diastolic pressure (LVEDP) was approximately regulated 5-10 mmHg by the alteration of balloon volume. Left ventricular systolic pressure (LVSP), Max dp/dt, and min dp/dt (±dp/dt max) were measure.^21^ Left ventricular developed pressure (LVDP) and rate pressure product (RPP) were calculated by following formula:

$$\begin{array}{c} \mathrm{LVDP}=\mathrm{LVSP}-\mathrm{LVEDP}\\ \mathrm{RPP}=\mathrm{LVDP}\times \mathrm{heart}\;\mathrm{rate} \end{array}$$

### Measurement of hypertrophy

After assessment of hemodynamic parameters using the Langendorff system, the hearts were removed and put in saline, then on a paper for assessment of the heart weight. Cardiac hypertrophy index (mg/g) was calculated from the total heart weight (mg) relative to total body weight (g) of the rat.^22^

### Measurement of antioxidant enzymes

After measurement of hypertrophy, 100 mg of heart tissue was frozen in liquid nitrogen and stored at -70°C. The tissue samples were homogenized in phosphate buffered saline (PBS; 50 mM at pH of 7.4) using a Homogenizer (Heidolph Silenterosher M, Germany), and centrifuged at 14000 g for 15 minutes. The assessment of enzyme levels including glutathione peroxidase (GPx), catalase (CAT) and superoxide dismutase (SOD) was performed on supernatant. GPx and SOD were measured using Randox kits (Randox Lab, UK) and CAT activity was evaluated using Zellbio kit (Zellbio Lab, Ulm, Germany).

### Statistical analysis

The results were indicated as mean and standard deviation (SD). In the present study, the normal distribution of the results was carried out by Kolmogorov-Smirnov analysis. One-way ANOVA and Least Significant Difference (LSD) test were used for comparison between the various groups. The comparison of pre and post metabolic in each group was performed by paired t-test using SPSS (version 16). A p < 0.05 was regarded statistically significant.

## Results

### Electrocardiographic parameters

**Figure 1:**
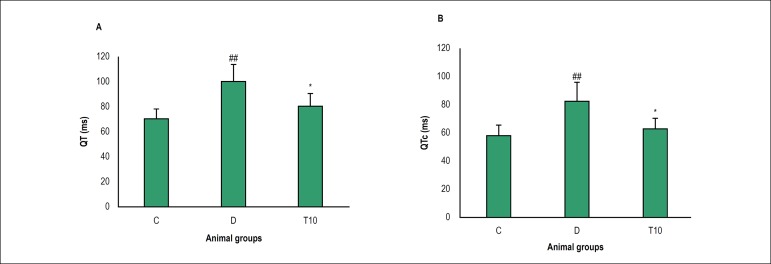
QT interval (a), QTc interval (b) values in control (C), diabetic (D) and diabetic treated with TMZ (10 mg/kg, T10) groups eight weeks after treatment in the rats. The results were presented as mean ± SD. ^##^ p < 0.01 compared to the control group, * p < 0.05 compared to the diabetic group.

The QT and QTc intervals significantly increased in the diabetic animals in comparison with the control group (100 ± 13.80 vs. 70 ± 8.34, 82.52 ± 13.03 vs. 58.4 ± 7.33, p = 0.007 and p=0.009, respectively). TMZ treatment was associated with a significant reduction in the QT and QTc intervals in comparison with the untreated diabetic rats (80 ± 10.69 vs. 100 ± 13.80, 63.11 ± 7.05 vs. 82.52 ± 13.03, p = 0.043 and p = 0.040, respectively, Figure 1). As shown in Table 1, the diabetic rats indicated a decrease in the heart rate compared to the control rats (198 ± 41.21 vs. 268 ± 27.99, p = 0.002). Obviously, the administration of diabetic group with TMZ significantly increased the heart rate compared to the untreated diabetic rats (263 ± 35.02 vs. 198 ± 41.21, p = 0.006).

**Table 1:** Hemodynamic parameters in the heart

Groups	C	D	T10	P value**D VS. C	P value**T10 VS. D
Heart rate (beats/min)	268 ± 27.99	198 ± 41.21	263 ± 35.02	0.002^##^	0.006**
LVSP (mmHg)	75 ± 20.91	60.78 ± 16.76	79.75 ± 10.16	0.041^#^	0.028*
LVDP (mmHg)	74.37 ± 18.76	56 ± 18.37	74.25 ± 9.93	0.030^#^	0.031*
RPP (mmHg)	14965 ± 5582	10184 ± 4589	14099 ± 3859	0.041^#^	0.049*
Max +dp/dt(mmHg)	2294 ± 255.27	1035 ± 370.33	1727 ± 410.60	< 0.001^###^	0.001**
Min -dp/dt (mmHg)	-1220 ± 229.09	-594.77 ± 210	-962 ± 194	< 0.001^###^	0.002**

(Mean ± SD, n = 8) in control (C), diabetic (D) and diabetic treated with TMZ (T10), (one-way ANOVA followed by LSD post hoc test). LVSP: left ventricular systolic pressure; LVDP: left ventricular developed pressure; RPP: rate pressure product.

### Markers of cardiac function

At the end of the experiment, LVSP, LVDP, ±dp/dt max and RPP were observed significantly lower in the diabetic group than control group. However, TMZ administration for 8 weeks was associated with a significant increase in these parameters in comparison with the untreated diabetic rats (Table 1).

### Effect of TMZ on myocardial hypertrophy

**Figure 2:**
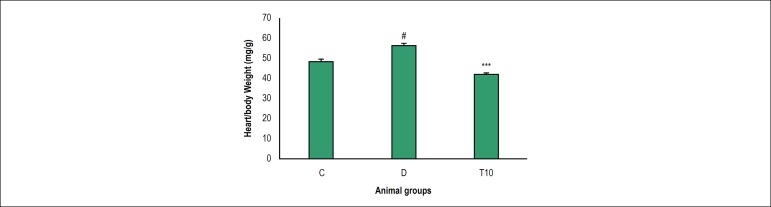
Hypertrophy value in control (C), diabetic (D) and diabetic treated with TMZ (T10) groups eight weeks after treatment in the rats. The results were presented as mean ± SD. ^#^ p < 0.05 compared with control group, ***p < 0.001 compared to the untreated diabetic group.

As indicated, the hypertrophy index increased significantly in the diabetic rats on 8 weeks compared to the control group (56.62 ± 6.50 vs. 48.62 ± 7.90, p = 0.039). According to our findings, in the diabetic rats, administration with TMZ remarkably decreased the hypertrophy index when compared to the diabetic rats (41.87 ± 7.50 vs. 56.62 ± 6.50, p < 0.001, Figure 2).

### Effect of TMZ on antioxidant enzymes

As indicated in Table 2, antioxidant enzymes, GPx, CAT and SOD significantly decreased in the heart of diabetic animals as compared to the control group (p < 0.001, p = 0.002, respectively). However, oral administration with TMZ was significantly improved GPx, CAT and SOD (p < 0.001, p < 0.049, respectively).

**Table 2:** Antioxidant enzymes activities

Groups	C	D	T10	P value D vs. C	P value**T10 vs. D
SOD (U/dl)	8.46 ± 1.51	5.86 ± 0.69	7 ± 1.54	0.002^##^	0.049*
CAT (U/ dl)	10.52 ± 0.60	1.90 ± 4.08	10.71 ± 0.50	0.002^##^	< 0.001***
GPx (U/ dl)	28.50 ± 2.67	13.22 ± 0.95	24.03 ± 1.73	< 0.001^###^	< 0.001***

(Mean ± SD, n = 8) in control (C), diabetic (D) and diabetic treated with TMZ (T10), (one-way ANOVA followed by LSD post hoc test). SOD: superoxide dismutase. CAT: catalase; GPx: glutathione peroxidase.

## Discussion

Our results indicated that alloxan injection significantly increased QT and QTc intervals and decreased heart rate, LVSP, LVDP, RPP, ±dp/dt max, and cardiac hypertrophy, SOD, GPx and CAT in the heart of the diabetic rats when compared with control group. However, treatment with TMZ was able to improve QT and QTc intervals, heart rate, hemodynamic parameters, SOD, CAT and hypertrophy significantly. Previous studies have demonstrated that diabetes is associated with the alterations of electromechanical and prolonged QTc interval in the heart.^23^

Diastolic and systolic dysfunctions are the earliest manifestations in the development of diabetic cardiomyopathy.^24^ The ±dp/dt max, LVSP, LVDP, RPP, cardiac diastolic and systolic indexes, are widely used to evaluate cardiac function. The alloxan-induced diabetic rats progressed cardiac dysfunction as demonstrated by a significant decrease in ±dp/dt LVSP, LVDP, RPP. TMZ treatment in turn improved each of these parameters.

In our model of type 1 diabetes, ECG indicated prolonged QTc, a finding that is consistent with previous studies. Treatment with TMZ significantly decreased these QT and QTc dispersions. This result is in agreement with previous reports which indicated that TMZ treatment improved QT prolongation in individuals with kidney disorders.^25,26^

In the present study, we also observed that diabetes led to bradycardia in the diabetic animals. It is revealed that in the diabetic rats heart rate tends to decrease after eight weeks.^27^ On the other hand, diabetes increases vagal tone and decreases sympathetic tone in diabetic rats.^28^ In addition, treatment with TMZ improves autonomic tone in individuals with acute coronary syndrome.^29^ Improved sympathetic and parasympathetic tone can partly explain the increased heart rate in the diabetic rats treated with TMZ.

Diabetic cardiomyopathy is associated with cardiac hypertrophy and dysfunction. High blood glucose and oxidative stress maybe considered to be critical factors that involved in hypertrophy and dysfunction of the heart.^30^ In the present study, the diabetic rats showed cardiac hypertrophy demonstrated by the increased heart wieght/body wieght ratio. Similar results have been indicated in previous studies.^31^ It is well established that, increased VLDL-c and decreased HDL-c levels can result in reduction in anti-oxidant defense system.^27^ In a previous study, it was indicated that the impairment of lipid profile levels in diabetic animals could be attributed to increased lipid breakdown and release of a large amount of free fatty acids.^17^ The released free fatty acids are susceptible to oxidation which result in decreased anti-oxidant level and anti-oxidant defense system.^32^ Increased level of fatty acid oxidation in the diabetic heart leads to lipid accumulation and cardiac hypertrophy.^33^ Reduction in fatty acid oxidation and oxidative stress by TMZ treatment can partly attribute to improvement of cardiac hypertrophy.

Previous studies have indicated that SOD level reduced in type 1 diabetes and it is mostly demonstrated that increased reactive oxygen species (ROS) negatively associated with the enzyme antioxidant values such as SOD and GPx.^34^ SOD quickly alters O_2_ to H_2_O_2_, which is further destroyed via GPx and CAT. The antioxidant enzyme levels are sensitive to the oxidative stress, and enhanced or reduced values have been indicated in various pathologies in which an increase of ROS is a cause or result of the disorder such as diabetes.^35,36^ In addition, superoxide anions and ROS have also been indicated to be contributed to cardiac hypertrophy resulted from various stimuli; therefore, SOD is a primary defense against oxidative stress that involves in the hypertrophy of the heart.^37^ Our findings indicated that SOD and CAT levels in hearts from TMZ treated diabetic rats was significantly higher than that in the untreated diabetic animals. GPx values was slightly but not significantly more in the hearts from TMZ treated diabetic animals compared to the diabetic rats.

Taken together, these findings indicated that the diabetic rats showed hypertrophy and dysfunction in the heart as well as increased cardiac oxidative damage in comparison with the control animals, showing that these undesirable factors are connected. TMZ probably improved these factors by antioxidant effects. Based on the results of present study, more studies require to be carried out to assessment mechanisms involved in the improvement of hypertrophy and cardiovascular disorders resulted from diabetes using TMZ treatment.

## Conclusions

All these observations show that TMZ treatment contributes to the improvement of impaired function and electrical activity as well as hypertrophy of the heart in diabetic cardiomyopathy in rats. Improvements observed in TMZ treatment is associated with decrease oxidative stress.
